# Structural basis of cell-surface signaling by a conserved sigma regulator in Gram-negative bacteria

**DOI:** 10.1074/jbc.RA119.010697

**Published:** 2020-02-26

**Authors:** Jaime L. Jensen, Beau D. Jernberg, Sangita C. Sinha, Christopher L. Colbert

**Affiliations:** Department of Chemistry and Biochemistry, North Dakota State University, Fargo, North Dakota 58108

**Keywords:** bacterial signal transduction, biophysics, metal homeostasis, protein structure, structural biology, X-ray crystallography, X-ray scattering, cell surface signaling, inner membrane sigma regulator, TonB-dependent transducer

## Abstract

Cell-surface signaling (CSS) in Gram-negative bacteria involves highly conserved regulatory pathways that optimize gene expression by transducing extracellular environmental signals to the cytoplasm via inner-membrane sigma regulators. The molecular details of ferric siderophore-mediated activation of the iron import machinery through a sigma regulator are unclear. Here, we present the 1.56 Å resolution structure of the periplasmic complex of the C-terminal CSS domain (CCSSD) of PupR, the sigma regulator in the *Pseudomonas capeferrum* pseudobactin BN7/8 transport system, and the N-terminal signaling domain (NTSD) of PupB, an outer-membrane TonB-dependent transducer. The structure revealed that the CCSSD consists of two subdomains: a juxta-membrane subdomain, which has a novel all-β-fold, followed by a secretin/TonB, short N-terminal subdomain at the C terminus of the CCSSD, a previously unobserved topological arrangement of this domain. Using affinity pulldown assays, isothermal titration calorimetry, and thermal denaturation CD spectroscopy, we show that both subdomains are required for binding the NTSD with micromolar affinity and that NTSD binding improves CCSSD stability. Our findings prompt us to present a revised model of CSS wherein the CCSSD:NTSD complex forms prior to ferric-siderophore binding. Upon siderophore binding, conformational changes in the CCSSD enable regulated intramembrane proteolysis of the sigma regulator, ultimately resulting in transcriptional regulation.

## Introduction

Cell-surface signaling (CSS)^3^ pathways allow Gram-negative bacteria to provide a rapid and efficient response to environmental stimuli through transcriptional activation. Key conserved components of CSS pathways are 1) an outer membrane transducer/transporter, which transduces the extra-cytoplasmic signal to the periplasm and also imports extracellular metabolites; 2) an inner membrane sigma regulator, also known as an anti-sigma factor, which transfers the signal from the periplasm to the cytoplasm; and 3) an extra-cytoplasmic function sigma factor that is released from the inner membrane to initiate expression of a target response gene (1). CSS systems are associated with biofilm formation, intercellular interactions, and release of virulence factors, in addition to metabolite transfer and regulation (2). One such CSS pathway involves iron import in Gram-negative bacteria. The best characterized CSS iron import systems are the ferric citrate (*fec*) transport system from *Escherichia coli*, the ferric pyoverdine (*fpv*) import system from *Pseudomonas aeruginosa*, and the ferric pseudobactin BN7/BN8 (*pup*) system from *Pseudomonas capeferrum* (formerly *Pseudomonas putida* WCS358). Each of these homologous pathways involves a TonB-dependent transporter/transducer, an inner membrane sigma regulator, and an extra-cytoplasmic function sigma factor (Table 1).

**Table 1:** **Protein components of the most well-studied CSS iron import systems from *P. capeferrum, E. coli, and P. aeruginosa***

	TonB-dependent transducer	Sigma regulator	Sigma factor
*P. capeferrum*	PupB*^a^*	PupR*^a^*	PupI
*E. coli*	FecA	FecR	FecI
*P. aeruginosa*	FpvA	FpvR	FpvI, PvdS

*^a^* This study.

Sigma regulators are central in the iron import CSS pathways. Sigma regulators are proteins of ∼325 amino acids consisting of three domains, 1) an N-terminal anti-sigma domain (3, 4), which regulates the sigma factor; 2) a single-pass transmembrane helix; and 3) a C-terminal periplasmic domain of ∼200 residues, responsible for interacting with the transducer (5, 6). The periplasmic domain of the sigma regulator FecR has been shown to interact with the N-terminal signaling domain (NTSD) of its cognate transporter/transducer, FecA (5, 7), and mutation of conserved hydrophobic residues to proline within this periplasmic domain disrupted binding to the NTSD (7). The structure of the periplasmic domain of sigma regulators has not been described.

Current studies suggest that signal activation involves regulated intramembrane proteolysis of the sigma regulator (6, 8–11). Siderophore uptake triggers a signal, presumably a protein interaction event between the transducer and the sigma regulator, which results in cleavage of the sigma regulator by an unidentified site-1 protease such as Prc, as shown for both FecR and FpvR, followed by intramembrane cleavage by a site-2 protease such as RseP (6, 8–11). Prc, a site-1 serine protease, was shown to proteolyze the periplasmic sigma regulator domain in IutY from *P. putida* KT2440, although fragments of IutY are present in non-CSS conditions (8). Alternatively, initial cleavage of the sigma regulator has also been proposed to include an autoproteolytic event via N-O acyl rearrangement through the nonconserved residues Gly^191^ and Thr^192^ of FoxR from *P. aeruginosa* (8, 12, 13). Therefore, the role of Prc in classical CSS pathways has not been fully established.

Here, we report the 1.56 Å resolution X-ray crystal structure of the periplasmic domain of the CSS sigma regulator, PupR, in complex with the NTSD of the transducer, PupB, revealing a unique fold and topological arrangement of domains. This is the first report describing the structure of the periplasmic region of a CSS sigma regulator, hereafter referred to as the C-terminal cell-surface signaling domain (CCSSD). The CCSSD comprises two subdomains: residues 110–238 that we call the C-terminal juxta-membrane subdomain (CJM) and residues 250–324, comprising a Secretin/TonB, short N-terminal subdomain (STN). The CJM has a novel-fold, whereas the STN is structurally homologous to the PupB NTSD. This structure, together with affinity pulldown assays, indicates that both subdomains are necessary to define the binding surface for the PupB NTSD. Furthermore, our biochemical and biophysical experiments demonstrate that the PupR CCSSD is highly unstable in the absence of PupB NTSD. Together, these results help to establish the molecular details of this cell-surface signaling interaction and provide a structural rationale for how CSS is triggered through the interaction of the sigma regulator with the outer membrane transducer.

## Results

### The PupR CCSSD comprises two subdomains, both of which are required for binding the PupB NTSD

**Figure 1.**
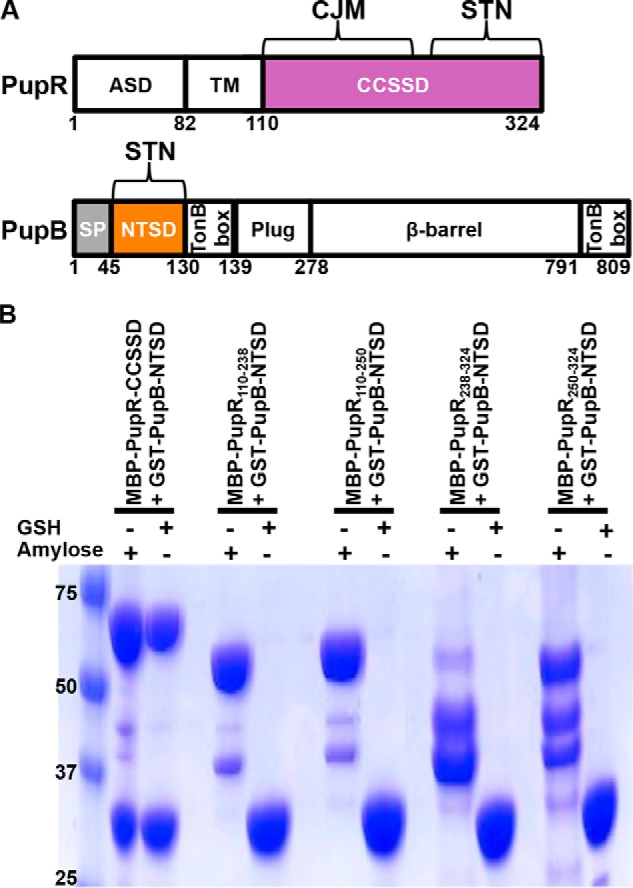
*A,* domain organization of PupR (an ASD, transmembrane region (*TM*), and CCSSD)) and PupB (a signal peptide (*SP*), NTSD, TonB box (region that interacts with the TonB complex), plug, β-barrel, and C-terminal TonB box)). Regions included in the expression constructs are colored. *B,* affinity pulldown assays to detect interaction of GST-tagged PupB NTSD and different MBP-tagged PupR CCSSD fragments as indicated. Equivalent aliquots of the clarified lysate from a co-expression of the two component proteins were applied to either amylose affinity agarose or GSH-Sepharose resins. Each resin was washed, then protein was eluted and analyzed by Coomassie-stained SDS-PAGE. The + sign *above* each lane indicates which resin was used for each experiment. The masses (kDa) of molecular weight markers are indicated in the *first lane*.

The domain boundaries of PupR, based on predictions of secondary structure using JPRED (14) and transmembrane helix(ces) using HHMTOP (15) are: a cytoplasmic N-terminal anti-sigma domain (ASD), comprising residues 1–82 (3); a single-pass transmembrane helix, residues 86–104; and a periplasmic CCSSD, residues 110–324 (Fig. 1*A*). The CCSSD has two potential subdomains: residues 110–238, which constitute a subdomain named the CJM subdomain, and a second subdomain, comprising residues 250–324, that belongs to the STN domain family (SMART accession number SM00965) (16, 17). However, when purified separately, these subdomains degrade rapidly and can only be individually purified as maltose-binding protein (MBP) fusion proteins, with the MBP-tagged STN still being very unstable.

**Figure 2.**
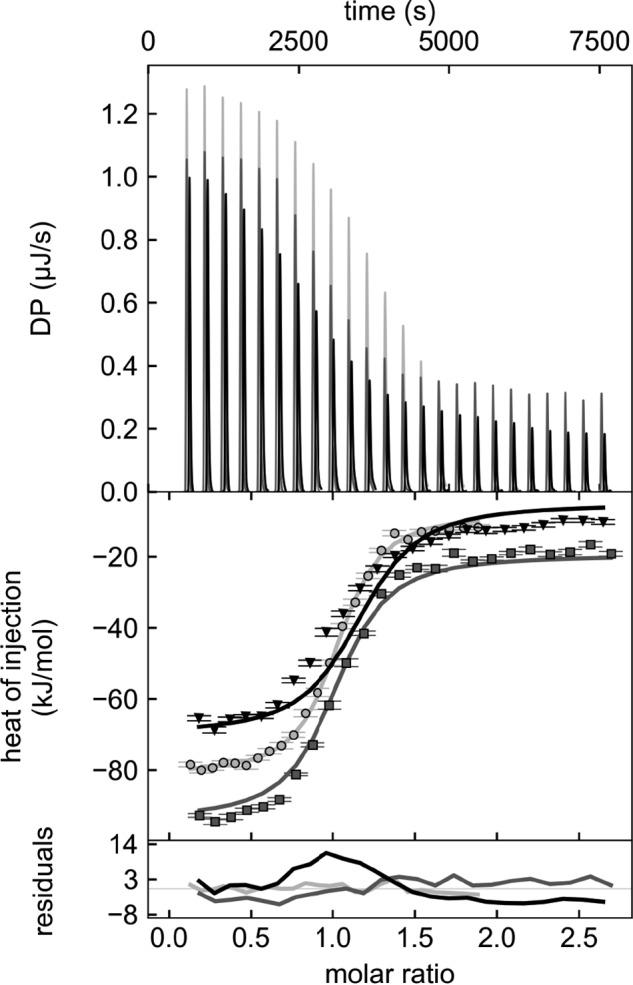
**Global analysis of ITC isotherms for PupR CCSSD titrated into PupB NTSD.** The heats of binding (*top panel*), the isotherms with the curves for the global model (*middle panel*), and residuals of the global model fit (*bottom panel*) for the triplicate experiments are shown in *black*, *gray*, and *light gray*.

PupB residues 45–130 comprise the NTSD (Fig. 1*A*) (18). The role of each CCSSD subdomain in binding the NTSD was delineated by affinity pulldown assays using MBP-tagged CJM (PupR^110–238^ or PupR^110–250^) or STN (PupR^238–324^ or PupR^250–324^) subdomains and GSH *S*-transferase (GST)-tagged NTSD (PupB^49–128^) fusion proteins. Although the complete CCSSD clearly binds to the NTSD (Fig. 1*B*), neither the isolated CJM nor STN subdomains associate with the NTSD (Fig. 1*B*). This indicates that individually, either the subdomains are insufficient for binding the NTSD, or that the subdomains are unfolded and binding-incompetent. Isothermal titration calorimetry (ITC) measurements indicate that the CCSSD and the NTSD bind in a 1:1 stoichiometry with an affinity (*K_D_*) of 0.69 μm with a 68.3% confidence interval of [0.42, 1.11 μm] (values in square brackets indicate a 68.3% confidence interval (±1 standard deviation) for the mean value presented) (Fig. 2, Table 2). Our binding model includes a local incompetent fraction parameter during isotherm analysis due to CCSSD precipitation during ITC measurements and presence of residual MBP. The local incompetent fraction range was 0–12.8% among the triplicate experiments.

**Table 2:** **Comparison of secondary structure content estimated from CD spectra analyses using CDPro and from DSSP assignments within PyMOL of the X-ray crystal structure**

Protein	CD analyses (N_res_)				X-ray structure (N_res_)			
	Helix	Strand	Coil + turn	Total	Helix	Strand	Coil + turn	Total*^a^*
CCSSD	16	89	113	218	31	112	76	219
NTSD	16	30	36	82	25	24	33	82
Complex	55	100	145	300	56	136	109	301

*^a^* Total number of residues indicates the full expressed protein, including any additional residues remaining after cleavage of affinity tags.

### Interaction of the PupR CCSSD with the PupB NTSD stabilizes the sigma regulator

**Figure 3.**
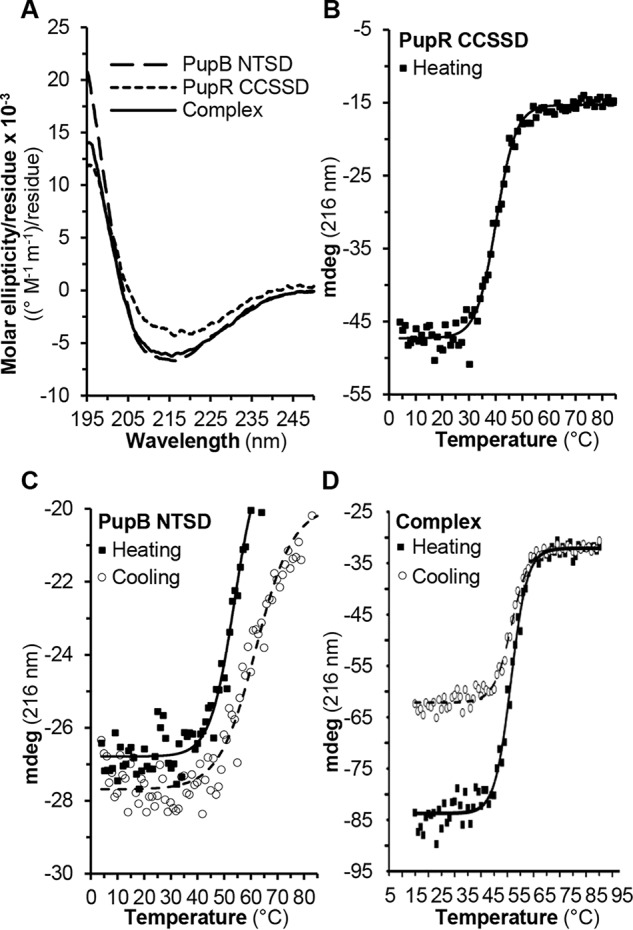
**CD spectra and melting curves.** *A,* CD spectra of PupB NTSD (*dashes*), PupR CCSSD (*dots*), and the complex (*solid*); *B,* PupR CCSSD melting curve; *C,* PupB NTSD melting curve; and *D,* the complex melting curve. Unfolding (heating; *black squares*) and refolding (cooling; *open circles*) data points are shown. The Boltzmann fits to the melting curves are shown.

Analyses of the circular dichroism (CD) spectra of the isolated CCSSD reveal it has significant secondary structure (Table 3, Fig. 3). The secondary structure content estimated from the CD spectra of the CCSSD:NTSD complex is comparable with the sum of secondary structure content estimated from the CCSSD and NTSD separately (Fig. 3*A*), suggesting that these domains do not undergo substantial secondary structure transitions upon binding (Table 3).

**Table 3:** **Thermodynamic parameters of the CCSSD:NTSD interaction as determined from ITC data using a global analysis in SEDPHAT** Mean values were determined from a global fit to a set of three ITC experiments.

	NTSD (μm)	CCSSD (μm)	LIF*^a^*	*K_d_* (μm)	Δ*H* (kJ/mol)	Δ*S* (J/mol·K)	Δ*G* (kJ/mol)
Set 1 (3 runs)	28	220	0.128	0.69 [0.42, 1.11]*^b^*	−73.99 [−80.99, −68.27]*^b^*	−138.83 [-158.94, −122.93]*^b^*	−33.990 [−35.196, −32.843]*^b^*
	27.5	220	0.00				
	42	235	0.051				

*^a^* Local incompetent fraction (LIF).

*^b^* Values in square brackets indicate a 68.3% confidence interval (±1 S.D.) for the mean value presented.

The thermal denaturation CD curve of the CCSSD, recorded at the spectral minima of 216 nm, indicates it has a melting temperature (*T_m_*) of 40.2 °C (Fig. 3*B*); however, the CCSSD precipitates during cooling renaturation. The thermal denaturation CD curves of the NTSD indicate it has a *T_m_* of 52.8 (heating) and 62 °C (cooling) (Fig. 3*C*) and its thermal denaturation is reversible. Strikingly, when the CCSSD is complexed with the NTSD, thermal denaturation of the whole complex is reversible and the *T_m_* of the complex increases to 51.4 or 52.9 °C for heating or cooling, respectively (Fig. 3*D*), demonstrating that binding of the NTSD stabilizes the CCSSD.

### The X-ray crystal structure of the PupR CCSSD:PupB NTSD reveals a unique fold and topological arrangement of subdomains within the PupR CCSSD

A high-quality electron-density map of the CCSSD:NTSD complex was obtained by single-wavelength anomalous diffraction (SAD) phasing using selenium atoms incorporated into the CCSSD. Refinement of the final atomic model was completed at 1.56 Å resolution with *R*_work_ = 15.0% and *R*_free_ = 18.3% (Table 4). The final Se-Met and native models include residues 111–323 of PupR, residues 49–128 PupB, 20 tartrate molecules, and 355 or 319 water molecules, respectively. The Se-Met and native proteins have practically identical structures, although there are some differences in interacting residues at the complex interface.

**Table 4:** **X-ray data collection, phasing, and refinement statistics for the PupR CCSSD:PupB NTSD complex** Values in parentheses pertain to the highest resolution shell.

	Native	Se-Met derivative
**Data collection**		
Beamline	24-ID-E	24-ID-C
Wavelength (Å)	0.9792	0.9792
Space group	*P*2_1_2_1_2_1_	*P*2_1_2_1_2_1_
Unit-cell parameters (Å, deg)	43.4, 44.6, 141.0 α, β, γ = 90	43.6, 44.7, 141.3 α, β, γ = 90
Resolution range (Å)	42.5–1.76 (1.767–1.761)	141.34–1.51 (1.53–1.51)
Total observations	190,024 (1895)	258,089 (2,816)
Unique observations	27,078 (2741)	43,910 (1,511)
Multiplicity	7.0 (6.9)	5.9 (1.9)
Completeness (%)	96.9 (99.3)	98.0 (70.2)
CC(1/2)	0.999 (0.966)	0.999 (0.765)
*R*_merge_*^a^* (%)	5.6 (26.8)	5.2 (40.1)
*R*_merge_ (anom, %)	–	4.5 (42.9)
Mean *I*/σ*I*	25.2 (6.8)	18.9 (1.6)
Data processing program	AutoPROC	HKL2000
**Refinement**		
Refinement program	PHENIX	PHENIX
Resolution range (Å)	42.5–1.76 (1.82–1.76)	42.6–1.56 (1.614–1.558)
Molecules per asymmetric unit	2	2
R_work_ (%)	16.0	15.3
R_free_ (%)	20.9	18.4
RMSD stereochemistry		
Bond lengths (Å)	0.011	0.018
Bond angles (deg)	1.21	1.96
No. of atoms	2670	2787
PupR CCSSD:PupB NTSD	2331	2412
Ligands (tartrate)	20	20
Waters	319	355
Total average *B* (Å^2^)	20.2	18.1
PupR CCSSD	19.1	16.1
PupB NTSD	23.4	18.6
Tartrate	23.5	20.0
Waters	28.8	27.4
Ramachandran plot (%)		
Preferred	98	98
Allowed	2	2
Outliers	0	0
**PDB code**	6OVK	6OVM

*^a^ R*_merge_ = Σ*_hkl_*Σ*_j_*|*I_hkl,j_* − 〈*I_hkl_*〉|/Σ*_hkl_*Σ*_j_I_hkl,j_*.

**Figure 4.**
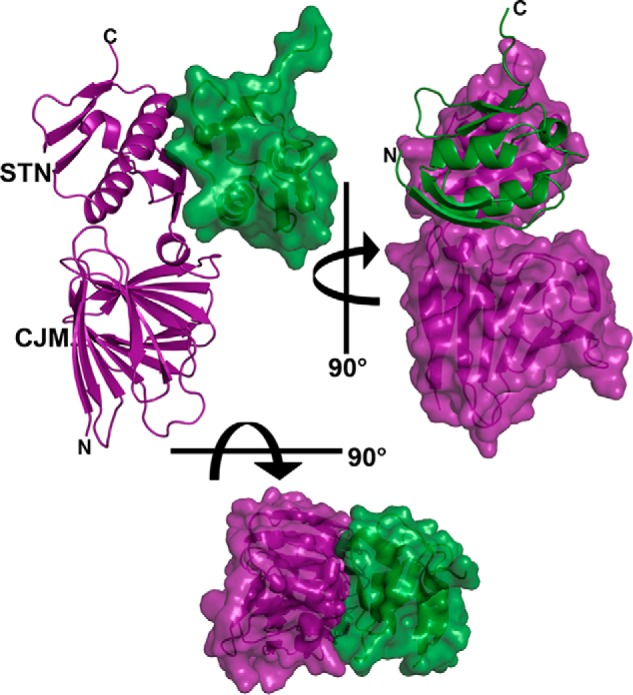
**The X-ray crystal structure of the PupR CCSSD:PupB NTSD complex.** Ribbon and transparent surface representations are colored *purple* for the PupR CCSSD and *green* for the PupB NTSD. The two CCSSD subdomains, the CJM and STN, are indicated.

**Figure 5.**
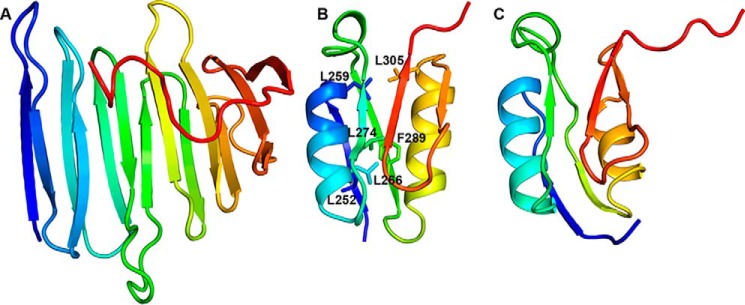
**Unique structural features of the PupR CCSSD.** All structures are displayed in ribbon, rainbow color-ramped from *blue* at the N terminus to *red* at the C terminus. *A,* the CJM subdomain has a novel all-β-fold. *B,* the STN subdomain of the CCSSD is shown with the conserved residues L252, L259, L274, L266, L305, and F289 from the “LLLV” region in stick. *C,* the PupB NTSD, displayed in a superimposable orientation to the STN subdomain in *B*.

The two subdomains are clearly delineated in the CCSSD structure (Fig. 4). The first subdomain, the CJM, comprising residues 110–238, has a novel all-β-fold that can be described as a twisted β-solenoid–like motif. A search through the PDB using DALI (19) did not reveal any structure with a Z-score >5.6 that has been previously described in literature. The CJM is comprised of two 7-stranded β-sheets linked by loops or β-arches: strands β2, β3, β6, β9, β10, β13, and β14 form an anti-parallel sheet, whereas strands β1, β4, β5, β7, β8, β11, and β12 form a mixed β-sheet (Fig. 5*A*). The hydrophobic packing of the side chains from the two β-sheets stabilizes the core of the CJM subdomain. As expected from sequence analyses, the second subdomain, comprising residues 250–324, belongs to the STN domain family (16, 17). It shares a common-fold, including two βαβ-repeat structural motifs, with the PupB NTSD (Fig. 5, *B* and C). A search of the SMART nonredundant database identifies over 8,000 proteins with STN domains, yet surprisingly, all these STN domains are arranged N-terminal to other domains within their respective proteins. Thus, the presence of an STN at the extreme C terminus of PupR (Figs. 1*A* and 4) represents a new architectural arrangement of this domain type. Sequence conservation suggests that the CCSSD-fold is common among periplasmic sigma regulator proteins (Fig. S1).

The CJM and STN are connected via an 11-residue linker that is primarily unstructured, apart from a single helical turn. The total buried surface area between the two subdomains is 821.9 Å^2^ with the interface stabilized by salt bridges between residues STN Arg^268^ and Asp^265^ to CJM Arg^192^ and Glu^159^, respectively (Fig. S2), and includes several partly or fully buried residues (Table S1).

### The PupR CCSSD:PupB NTSD interaction interface

The PupB NTSD shares 37.1% sequence identity with the PupA NTSD, 30.5% sequence identity with the FecA NTSD, and 28.4% sequence identity with the FpvA NTSD. As expected, the PupB NTSD structure in the CCSSD:NTSD complex is similar to the *P. capeferrum* PupA NTSD and *E. coli* FecA NTSD structures, determined using NMR (20, 21), and found in the structures of the complete *P. aeruginosa* FpvA transducer (22, 23). These NTSDs superimpose with root-mean-square deviations (RMSD) ranging from 1.29 to 2.58 Å over 72–80 Cα atoms.

The interface between the CCSSD and the NTSD has a substantial buried surface area of ∼1438.6 Å^2^ and involves residues from the linker, β17 and α2 of the CCSSD and α1 and β2 of the NTSD (Fig. 4). The interface is stabilized by salt bridges between NTSD His^72^ and Glu^83^ to CCSSD Glu^292^ and Arg^284^, respectively (Fig. S3, *A* and *B*), as well as an extensive hydrogen-bonding network (Fig. S3, *C–E*, Table S2). Hydrophobic interactions at the interface include two extensively buried residues, NTSD Leu^74^ (84% buried) and CCSSD Met^251^ (98% buried) (Fig. S3, *F* and *G*).

Previously, residues 247–268 within the periplasmic domain of the homologous sigma regulator, FecR, were named the LLLV region as this region includes conserved leucine and valine residues (Fig. S1) (7). Mutation of these conserved hydrophobic residues to proline was shown to abrogate binding to the FecA NTSD (7). Our structure shows that this LLLV region corresponds to the hydrophobic core of the PupR STN subdomain (Fig. 5*B*) and does not directly mediate the interaction with the NTSD. Rather, our structure indicates that these residues are essential for the structural integrity of the STN and that mutation of these residues to proline likely disrupts secondary structure and causes unfolding of the subdomain, preventing it from binding to the NTSD.

### Small angle X-ray scattering coupled to size exclusion chromatography (SEC-SAXS) indicates the PupR CCSSD is partially flexible

**Figure 6.**
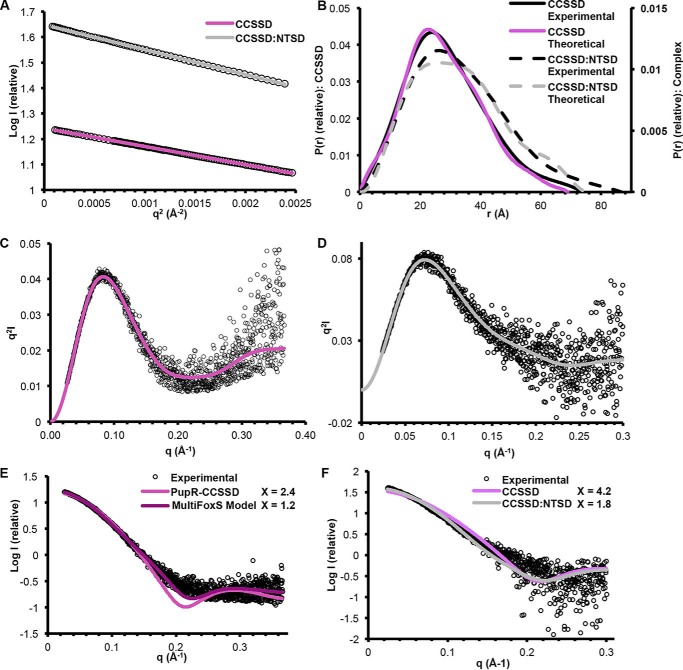
**SEC-SAXS analysis of the CCSSD and CCSSD:NTSD complex.** *A,* Guinier plot of the low *q* region. *B,* distance distribution *P(r*) for the experimental data (*black lines*), the theoretical curve calculated from the CCSSD crystal structure (*purple line*), and the CCSSD:NTSD complex (*gray dashed line*). Kratky plots of the (*C*) CCSSD and (*D*) the CCSSD:NTSD complex are shown. *E,* experimental scattering profile for the CCSSD, fit with the theoretical scattering profiles calculated from the rigid crystal structure of the CCSSD only (*purple*) and the flexible model derived from MultiFoxS, generated by structural conformation sampling (*dark purple*). *F,* experimental scattering profile for the complex, fit with the theoretical scattering profiles calculated from crystals structures of the CCSSD only (*purple*) and the CCSSD:NTSD complex (*gray*). χ values for each fit are indicated.

SEC-SAXS was used to determine and compare low-resolution structure and solution properties such as molecular mass and oligomeric states of the CCSSD and CCSSD:NTSD complex (Fig. 6, Table S3), and were performed concurrently with crystallographic experiments. Given the instability of the CCSSD alone in solution and secondary structure estimates from CD analysis, we hypothesized that the CCSSD is conformationally heterogeneous with multiple orientations between the subdomains when not bound to the NTSD. Linearity of the Guinier plot in the 0 < *q* < 0.003 Å verifies the absence of aggregation in the samples (Fig. 6*A,* Fig. S4). The radius of gyration (*R_g_*), calculated from the Guinier region (Fig. 6*A*), is 22 Å for the CCSSD and 26 Å for the CCSSD:NTSD complex, whereas the distance distribution function, *P*(*r*), indicates a *D*_max_ of 75 Å for the CCSSD and 87 Å for the complex (Fig. 6*B*). These values are in agreement with the theoretical *R_g_* calculated from a CCSSD-only model, and for the CCSSD:NTSD complex crystal structure. The Kratky plots indicate that both samples are partially flexible in solution (Fig. 6, *C* and *D*). The molecular mass of the CCSSD, estimated from the SAXS data is 23–29 kDa (theoretical mass = 24 kDa), indicating that the CCSSD is monomeric in solution (Fig. 6*E*, Table S3). The molecular mass of the CCSSD:NTSD complex, estimated from the SAXS data is 33–40 kDa (theoretical mass = 32.3 kDa), suggesting that the primary species in solution is a 1:1 complex (Fig. 6*F*, Table S3), consistent with the crystal structure and ITC data.

SAXS is also useful for evaluating the internal flexibility of multidomain proteins. The experimental SAXS curve of the CCSSD exhibited weak agreement with the theoretical curve calculated from the CCSSD crystal structure alone (Fig. 6*E*). The possibility of conformational heterogeneity of the PupR CCSSD in solution was explored using MultiFoxS to generate ∼10,000 conformers, maintaining the CJM and STN subdomains of the CCSSD as rigid bodies and defining residues 232–250 as a flexible linker. The experimental data best fit a two-state model, wherein the predominant conformation has a *R_g_* of 21.7 Å and comprises 87% of the solution state, and the secondary species has a *R_g_* of 18.7 Å and is sampled in 13% of conformations. The predominant species of the best-fitting conformers from each model improved the χ value to 1.24, and significantly improved the goodness of fit around *q* = 0.2 Å^−1^, indicating structural flexibility between the two subdomains of the CCSSD (Fig. S5). Similarly, whereas the scattering curve calculated from the complex fits better than that for the CCSSD alone, the fit of the complex is not perfect (Fig. S6), suggesting there could be inter-subdomain or inter-protein flexibility not accounted for by the crystal structure (Fig. 6*F*). MultiFoxS was used to assess various regions of potential flexibility and only marginally improved the fit (*X* = 1.2–1.8).

### Confirmation of the PupR CCSSD:PupB NTSD interaction interface

**Figure 7.**
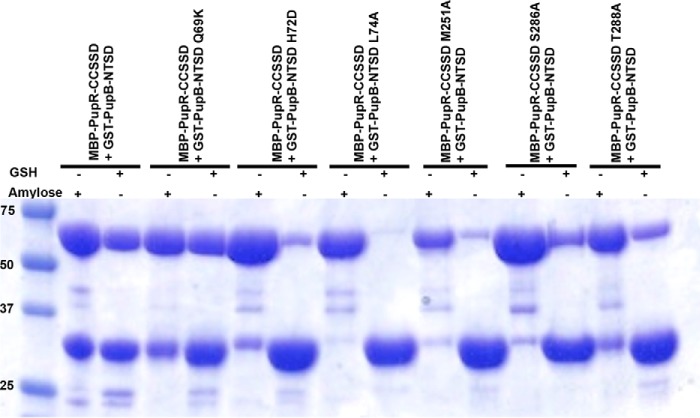
**Affinity pulldown assays to detect interaction between different GST-tagged PupB NTSD and MBP-tagged PupR CCSSD mutants.** Wild-type interaction between the PupR CCSSD and the PupB NTSD (*second* and *third lanes*). Residues stabilizing the PupR CCSSD:PupB NTSD interface were mutated as follows PupB Q69K (*fourth* and *fifth lanes*), PupB H72D (*sixth* and *seventh lanes*), PupB L74A (*eighth* and *ninth lanes*), PupR M251A (*10th* and *11th lanes*), PupR S286A (*12th* and *13th lanes*), and PupR T288A (*14th* and *15th lanes*). The Coomassie-stained SDS-PAGE gel is shown. The + sign *above* each lane indicates which affinity resin was used for each experiment, as in Fig. 1. The masses of molecular weight markers are indicated in the first lane.

The importance of key residues at the complex interface identified from the structure was qualitatively assessed by generating the following point mutations: NTSD residues Q69K, H72D (Fig. S3*A*), and L74A (Fig. S3*G*), and CCSSD residues M251A (Fig. S3*F*), S286A (Fig. S3*D*), and T288A (Fig. S3*E*). Residues Gln^69^ and His^72^ were mutated to the corresponding residues of the homologous, but signaling incompetent, PupA NTSD. The pulldown assays show that the H72D, L74A, and M251A mutations completely disrupt the CCSSD:NTSD interaction, whereas S286A and T288A appear to limit, but not completely abrogate, the interaction (Fig. 7).

Interestingly, a polar interaction linking NTSD His^72^ and CCSSD Glu^292^ is critical for interaction between the two proteins, but the atoms involved are variable. In the native crystal structure, the Nϵ2 of the His^72^ imidazole ring forms a salt bridge with Glu^292^ Oϵ1 and Oϵ2 (Fig. S4*A*). However, in the Se-Met–derivative crystals, the interaction is indirect, with the Glu^292^ side chain being replaced by a bridging water molecule that links the Nϵ2 of the His^72^ imidazole ring to the backbone amide of Leu^291^, the backbone amide of Glu^292^, and the backbone carbonyl of Gly^250^. The Glu^292^ side chain adopts a different conformer with the closest atom, Oϵ2, shifting 4.7 Å from His^72^. These results suggest that in addition to the complementary surfaces, a polar interaction at this position is critical to the interaction.

## Discussion

Our structure of the CCSSD:NTSD complex and our biophysical data answer several outstanding questions about the mechanism of CSS and help resolve conflicting hypotheses for the interaction between a sigma regulator and a TonB-dependent transducer. Our results reveal the CCSSD is comprised of two structured subdomains, the CJM and STN, which are linked by an 11-residue, conformationally-flexible linker. Furthermore, our structure and pulldown assays using various PupR CCSSD truncations indicate that the CJM and STN are both required for binding the PupB NTSD *in vitro*. Notably, PupR STN residues analogous to the FecR LLLV region, comprising residues 247–268, that were previously reported to be critical for binding to the FecA NTSD (7), correspond to the PupR STN subdomain hydrophobic core essential for structure integrity (Fig. 5) and do not directly mediate the interaction with the NTSD.

In contrast to the information provided here, a recent NMR study investigating the interaction of the C-terminal domain (CTD) of sigma regulator HasS with the NTSD of its cognate transducer HasR, members of the heme acquisition system (Has) of *Serratia marcescens*, suggests that the HasS CTD is partially disordered and contains a region that may interact with the inner membrane (24). However, the HasS CTD is analogous to the structured STN subdomain defined here. Our studies show that the STN subdomain is unstable in the absence of the CJM, and when not bound to the NTSD. Consistent with our observations, purification of the HasS CTD involved refolding of protein expressed into inclusion bodies. In the same study, chemical shift changes on the HasR NTSD were thought to indicate a “disordered wrapping mode” brought about by interaction with a partially disordered region of HasS (24). Our CD and SAXS data both indicate that whereas the PupR CCSSD in solution displays some flexibility in the 11-residue linker between the CJM and STN subdomains, the domain is largely folded even in the absence of NTSD binding. CD analyses of the isolated CCSSD in solution indicates the secondary structure content estimated is comparable with that of the CCSSD crystallized in complex with the NTSD. Indeed, comparison of secondary structure content of the isolated CCSSD and NTSD estimated via CD to that of the CCSSD:NTSD crystal content confirms that there are no dramatic changes in secondary structure upon complex formation. Last, α2 of the STN subdomain of the CCSSD packs against the NTSD. Hence, it is unlikely this region interacts with the inner membrane. Thus, our data appears to preclude the proposed disordered wrapping mode for association, and instead demonstrates that both the CCSSD and NTSD are ordered, and identifies the specific structural elements of each domain responsible for the interaction.

NTSD residues involved in the interaction are on a surface defined by α2 and β2 (PupB residues 60–80). Our site-directed mutagenesis of residues in this region, particularly His^72^ and Leu^74^, confirm the role of this region in binding. This is in contrast to previous studies with homologous NTSDs that suggest a region defined by the C terminus of α1 and the β3-α2 loop (5, 25, 26), which does not map to the CCSSD:NTSD interface in our crystal structure, is involved in interaction with the sigma regulator.

**Figure 8.**
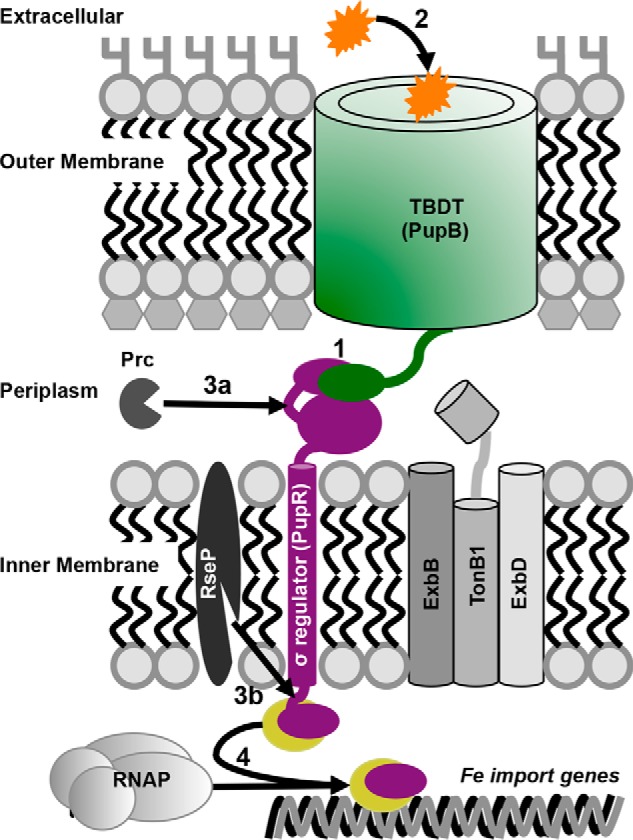
**Schematic of the proposed CSS activation model.** The proposed model starts with *1*) the CSS system being primed by the TBDT NTSD:CCSSD sigma regulator interaction that stabilizes the sigma regulator; *2*) ferric siderophore binding triggers signals for, *3*), *a* and *b,* regulated intramembrane proteolysis, resulting in *4*) release of the sigma regulator:sigma factor complex to activate transcription of iron import genes.

Finally, data showing activation of CSS by regulated intramembrane proteolysis indicate fragments of sigma regulators are present even under nonsignaling conditions (6, 8). Our research on the PupR sigma regulator provides a rationale for this phenomenon. It demonstrates that the CCSSD alone is highly dynamic and consequently, sensitive to proteolysis, but is stable when in complex with the NTSD. Therefore, until a transducer is located and bound, and the CCSSD stabilized, it may be nonspecifically proteolyzed. Together, our data leads us to propose a new model for the mechanism of the sigma regulator in CSS: this CSS system may be “primed” for activation, meaning the CCSSD must be stabilized by interacting with the NTSD so that it cannot be nonspecifically degraded (6, 8) (Fig. 8). Binding of siderophore to PupB induces conformational changes in the CCSSD:NTSD complex, causing the CCSSD to be recognized by a site-1 protease such as Prc, leading to initiation of regulated intramembrane proteolysis and subsequent cleavage by a site-2 protease (RseP) to release the ASD:sigma factor complex (Fig. 7).

## Materials and methods

### Cloning of PupR CCSSD constructs

Potential PupR domains were identified using secondary and tertiary structure predictions. Five expression constructs were made, comprising PupR residues 110–324, 110–238, 110–250, 238–324, or 250–324 cloned separately between NcoI and XhoI sites of the pMBP-Parallel1 vector (27).

### Protein expression and purification of PupB NTSD, MBP-tagged PupR CCSSD, and PupR CCSSD

The PupB NTSD was purified as described previously (18).

Chemically-competent *E. coli* C41(DE3) cells (Lucigen) were transformed using the pMBP-Parallel1-PupR CCSSD (PupR residues 110–324) plasmid for purification of MBP-tagged PupR CCSSD or PupR CCSSD. Transformed cells were grown at 37 °C in LB medium supplemented with 100 μg/ml of ampicillin to an OD_600_ of 0.7–0.9, and expression was induced with 0.5 mm isopropyl 1-thio-β-d-galactopyranoside at 20 °C for 20 h. Cells were harvested by centrifugation and stored at −80 °C. At each subsequent stage of purification, protein purity was analyzed by SDS-PAGE and protein concentration determined by absorbance at 280 nm using the molar extinction coefficient ϵ_280_ = 29,450 m^−1^ cm^−1^ and a theoretical molecular weight of 24,067 g/mol.

The cell pellet was thawed and resuspended in chilled lysis buffer (25 mm HEPES, pH 7.5, 400 mm LiCl, 10% glycerol, 2 mm DTT), then lysed with a Nano DeBEE homogenizer (BEE International). The crude extract was clarified by centrifugation at 20,000 × *g* for 45 min. The clarified supernatant was loaded onto amylose affinity resin equilibrated with lysis buffer, washed with lysis buffer, and fusion protein eluted with lysis buffer containing 20 mm maltose. Elution fractions were pooled and concentrated with a 30-kDa MWCO centrifugal filter unit (Millipore). The final step was SEC over a 16/60 Superdex 200 column (GE Lifesciences) equilibrated with lysis buffer without DTT. Fractions containing pure, homogeneous MBP-tagged PupR CCSSD were pooled, concentrated to 18 mg/ml, flash frozen in liquid nitrogen, and stored at −80 °C. MBP-tagged PupR CJM and MBP-tagged PupR STN were purified in a manner similar to the MBP-tagged PupR CCSSD.

PupR CCSSD was expressed and purified by amylose affinity chromatography, as for MBP-tagged PupR CCSSD. However, instead of eluting the purified fusion protein from the column, it was subjected to on-column cleavage by addition of recombinant tobacco etch virus protease in a 1:10 mass ratio, followed by a 16-h incubation at 4 °C, which yielded a 219-residue product, comprising PupR residues 110–324, preceded by a 4-residue (GAMG) cloning artifact. Cleaved PupR CCSSD was washed off the column and contaminating MBP removed by a second pass over equilibrated amylose resin. The PupR CCSSD was concentrated using a 10-kDa MWCO Millipore centrifugal filter unit prior to SEC, performed as described for MBP-tagged PupR CCSSD. Fractions containing PupR CCSSD were pooled, concentrated to 10 mg/ml, and stored at −80 °C. Final protein purity was estimated to be ∼90% by SDS-PAGE stained with Coomassie Blue (28) as it had some MBP contamination.

### Preparation of selenomethionine-derivatized PupR CCSSD

Selenomethionine (Acros Organics)-derivatized PupR CCSSD was expressed using a modified protocol involving methionine synthesis suppression (29, 30). *E. coli* C41(DE3) cells transformed with pMBP-Parallel1-PupR CCSSD were grown at 37 °C to saturation in 3 ml of LB medium with 100 μg/ml of ampicillin, then transferred to pre-warmed M9 minimal medium containing 2 mm MgSO_4_, 0.1 mm CaCl_2_, 0.4% (w/v) glucose, and 100 μg/ml of ampicillin and incubated at 37 °C. Once the OD_600 nm_ reached 1.0, the medium was supplemented with Se-Met, Lys, Thr, Phe, Leu, Ile, and Val; and the temperature lowered to 20 °C. Protein expression was induced with 0.5 mm isopropyl 1-thio-β-d-galactopyranoside for 18 h. Purification of Se-Met PupR CCSSD was performed as described for native protein. The molecular mass of the final protein samples and Se-Met incorporation were confirmed by electrospray ionization MS.

### Co-expression and affinity pulldown assays of PupR CCSSD:PupB NTSD complexes

*E. coli* BL21(DE3)pLysS cells were co-transformed with pMBP-Parallel1-PupR CCSSD and pET41-GST-PupB NTSD. Co-transformants were selected by growing on LB agar medium containing 100 μg/ml of ampicillin and 15 μg/ml of kanamycin. Co-expression followed the same purification procedure as for the individual proteins. Harvested cells were lysed and cell debris pelleted by centrifugation. The clarified supernatant was divided into two equal aliquots and combined with either 5 ml of amylose resin or 5 ml of GSH-Sepharose resin. The columns were incubated for 30 min at 4 °C. Each column was washed with 10 column volumes of lysis buffer, then eluted with lysis buffer + 20 mm maltose or lysis buffer + 15 mm GSH as appropriate. Total protein content was determined by Bradford assay, and 20 μg of protein were loaded onto a 4–20% TGX SDS-PAGE gel (Bio-Rad). Gels were stained with Coomassie Blue and qualitatively analyzed for protein association. This protocol was repeated for all pulldown analyses. The identity of the proteins in the pulldown assays was confirmed by Western blotting, using commercially available anti-MBP-HRP (New England Biolabs) or anti-GST-HRP (GE Healthcare) antibodies.

### CD spectroscopy and thermal denaturation of PupR CCSSD, PupB NTSD, and PupR CCSSD:PupB NTSD

PupR CCSSD, PupB NTSD, or PupR CCSSD:PupB NTSD samples were dialyzed in 10 mm potassium phosphate, pH 6.8, 100 mm (NH_4_)_2_SO_4_ overnight at 4 °C and diluted to 50 μm (0.204 mg/ml). Continuous scanning CD spectra were measured at 4 °C between 180 and 250 nm using a Jasco J-815 spectrometer with a PFD-425S Peltier cell holder and a 1-mm quartz cell. The spectra were buffer subtracted, and the secondary structure content estimated using CONTIN and CDSSTR, within the CDPro software suite (31).

CD melting and re-folding curves were recorded at 216 nm with 50 μm PupR CCSSD, PupB NTSD, or PupR CCSSD:PupB NTSD by increasing the temperature from 10 to 85 °C in 1 °C increments with a slope of 1 °C/min. Protein unfolding was monitored during both heating and cooling. Melting temperatures were determined by fitting a standard Boltzmann sigmoidal curve to the ellipticity in Origin 8 (OriginLab Corp., Northampton, MA). The final melting temperature was defined as the inflection point after fitting.

### ITC to quantify affinity of MBP-tagged PupR CCSSD binding to PupB NTSD

ITC was performed using a Low Volume Nano ITC (TA Instruments). Purified proteins were loaded into separate dialysis cassettes, and co-dialyzed against 25 mm HEPES, pH 7.5, 400 mm LiCl, 10% glycerol. All ITC experiments were performed at 15 °C, with 25 injections of 2 μl each. MBP-tagged PupR CCSSD, concentrated to 220–235 μm, was titrated into 27.5–40 μm PupB NTSD. Titrations were repeated in triplicate. The values from a buffer-into-buffer titration were subtracted from the values of the protein-into-protein titration during analysis. Data were analyzed with either NanoAnalyze (TA Instruments) with an independent, single-site model, or NITPIC (32) for data integration, followed by data processing with SEDPHAT (33–36) and plotting of isotherms in GUSSI (34). Processing included data refinement considering the local incompetent fraction as a function of the concentration compensation factor (37). To control for possible nonspecific interactions between MBP and PupB NTSD, 186–196 μm MBP was titrated into 45–57 μm PupB NTSD and analyzed.

### Crystallization, data collection, and structure solution of the PupR CCSSD:PupB NTSD complex

PupR CCSSD and PupB NTSD were combined in a 1:1 molar ratio. The MCSG crystallization suite (Anatrace) was used to identify initial crystallization conditions. Reproducible crystals were grown by sitting drop vapor diffusion in 200 mm sodium tartrate or Na-K tartrate, 20–25% (w/v) PEG 3350. Single crystals were cryoprotected with MiTeGen CryoOil and flash-frozen.

Diffraction data were collected at NE-CAT beamlines 24-ID-E and 24-ID-C at the APS under cryogenic conditions (∼100 K). The native PupR CCSSD:PupB NTSD diffraction data set was processed using autoPROC (38) with components POINTLESS (39) for space group determination, MOSFLM (40) for indexing, and XDS (41) and SCALA (42) for scaling. Diffraction data from a single, orthorhombic crystal of Se-Met PupR CCSSD:PupB NTSD was processed with HKL2000 (43). The structure was determined to 1.6 Å by SAD phasing. Three of the four selenium sites per PupR CCSSD monomer were located, and initial phasing was performed using AutoSol in PHENIX (44). Initial electron density maps were interpreted by automated model building using AutoBuild (45).

Refinement was carried out in PHENIX (46) with iterative model building in COOT (47). The Se-Met PupR CCSSD structure was used for molecular replacement (MR) against the native data set at 1.76 Å using Phaser-MR (48) followed by AutoBuild (45). Automated TLS group determination (49) and individual atomic B-factors were used during refinement in PHENIX for both the Se-Met and native structures. The quality of the diffraction data and final refined structures are summarized in Table 3.

Model validation was performed using MolProbity (50) and the PDB Validation Server (SCR_018135). Analyses of surface areas, protein interfaces, assemblies, and interactions were performed using the PISA server (SCR_015749) (51). RMSD comparisons were carried out in PyMOL (52). The DALI protein structure comparison server (SCR_013433) using DaliLite v.5 was used to identify the fold, family, and superfamily of each subdomain of the structure (19).

### SEC-SAXS measurements and analysis

SAXS data were recorded in tandem with SEC at BioCAT (beamline ID-18) at the Advanced Photon Source. Experimental details and structural parameters are summarized in Table S3. Prior to measurements, an inline Superdex 200 Increase 10/300 column was equilibrated with 25 mm HEPES, pH 7.5, 400 mm LiCl, 10% (v/v) glycerol. PupB NTSD and PupR CCSSD were combined at a 1:1 molar ratio and incubated for 30 min at room temperature prior to loading. 800 μm complex or 400 μm PupR CCSSD alone were injected onto the SEC column with a flow rate of 0.6 ml/min, and scattering data recorded from a 1s exposure every 3 s at ambient temperature. Scattering data were collected at a wavelength of 1.03 Å (∼12 keV), covering a momentum transfer range (*q*) of 0.004–0.36 Å^−1^, using a Pilatus 3 1M detector at a distance of 3.5 m from the sample. Scattering data were normalized to the incident X-ray beam, and scattering from the SEC buffer was subtracted with Igor Pro and BioCAT beamline extension programs.

SAXS data analyses were performed using the ATSAS suite (53). PRIMUS was used for data merging, calculating the *R_g_* with a Guinier approximation, and evaluating protein order by the Kratky plot (54). The absence of protein aggregation was validated by examining the linearity of the Guinier region. The pair distribution function, *P*(*r*), and maximum particle dimension, *D*_max_, were determined in GNOM (55). Molecular weight was determined using the SAXS Molecular Weight webserver (SCR_018137) (56). Theoretical scattering of the crystal structures was computed and fitted with the experimental data using CRYSOL (57).

To evaluate the flexibility of the PupR CCSSD linker, the HingeProt (58) webserver (SCR_018136) was used to identify the optimal linker from the CCSSD structure. It identified two possibilities, residues 239–250 or 232–250, which were input as flexible for multistate modeling with MultiFoXS (59). The models with the lowest χ-squared values and deviations from experimental data were identified. Additionally, EOM 2.0 (60) was utilized to generate PupR CCSSD flexible conformers that align with the SAXS profile, using the two subdomains and full PupR CCSSD sequence as the input files. Similarly, the CCSSD:NTSD SAXS scattering curve was evaluated using MultiFoXS, using various residues as being potentially disordered.

### Site-directed mutagenesis of PupR CCSSD and PupB NTSD

Point mutations of WT PupR CCSSD or WT PupB NTSD were created from the expression vectors described above using a QuikChange II kit (Agilent). DNA sequencing verified the gene sequences of the mutant plasmids used for transformation.

### Data availability

The atomic coordinates and structure factors have been deposited into the Protein Data Bank (http://www.rcsb.org)^4^ under PDB entries 6OVM and 6OVK. SAXS data has been deposited into the Small Angle Scattering Biological Data Bank (SASBDB; https://www.sasbdb.org)^4^ under entries SASDGA5 and SASDGU5 (61).

## Author contributions

J. L. J., B. D. J., S. S., and C. L. C. conceptualization; J. L. J. resources; J. L. J., B. D. J., and C. L. C. data curation; J. L. J., B. D. J., and C. L. C. formal analysis; J. L. J., B. D. J., and C. L. C. validation; J. L. J., B. D. J., and C. L. C. investigation; J. L. J. and C. L. C. visualization; J. L. J. methodology; J. L. J. writing-original draft; J. L. J., B. D. J., S. S., and C. L. C. writing-review and editing; S. S. and C. L. C. supervision; S. S. and C. L. C. funding acquisition; C. L. C. project administration.

## Supplementary Material

Supporting Information

## Footnotes

4 Please note that the JBC is not responsible for the long-term archiving and maintenance of this site or any other third party hosted site.

## Footnotes

3 The abbreviations used are:

CSS: cell-surface signaling
CCSSD: C-terminal cell-surface signaling domain
NTSD: N-terminal signaling domain
CTD: C-terminal domain
CJM: C-terminal juxta-membrane domain
STN: Secretin/TonB short N-terminal subdomain
ASD: anti-sigma domain
MBP: maltose-binding protein
GST: glutathione *S*-transferase
ITC: isothermal titration calorimetry
Se-Met: selenomethionine
RMSD: root-mean-square deviation(s)
SEC-SAXS: size exclusion chromatography small-angle X-ray scattering
NTD: N-terminal domain
HRP: horseradish peroxidase
SAD: single-wavelength anomalous dispersion
MR: molecular replacement
fec: ferric citrate.

## References

[1] Noinaj N., Guillier M., Barnard T. J., and Buchanan S. K. (2010) TonB-dependent transporters: regulation, structure, and function. Annu. Rev. Microbiol. 64, 43–60 10.1146/annurev.micro.112408.134247 20420522 PMC3108441

[2] Llamas M. A., Imperi F., Visca P., and Lamont I. L. (2014) Cell-surface signaling in *Pseudomonas*: stress responses, iron transport, and pathogenicity. FEMS Microbiol. Rev. 38, 569–597 10.1111/1574-6976.12078 24923658

[3] Jensen J. L., Balbo A., Neau D. B., Chakravarthy S., Zhao H., Sinha S. C., and Colbert C. L. (2015) Mechanistic implications of the unique structural features and dimerization of the cytoplasmic domain of the *Pseudomonas* sigma regulator, PupR. Biochemistry 54, 5867–5877 10.1021/acs.biochem.5b00826 26313375 PMC4701049

[4] Edgar R. J., Xu X., Shirley M., Konings A. F., Martin L. W., Ackerley D. F., and Lamont I. L. (2014) Interactions between an anti-sigma protein and two sigma factors that regulate the pyoverdine signaling pathway in Pseudomonas aeruginosa. BMC Microbiol. 14, 287 10.1186/s12866-014-0287-2 25433393 PMC4256889

[5] Enz S., Mahren S., Stroeher U. H., and Braun V. (2000) Surface signaling in ferric citrate transport gene induction: interaction of the FecA, FecR, and FecI regulatory proteins. J. Bacteriol. 182, 637–646 10.1128/JB.182.3.637-646.2000 10633096 PMC94325

[6] Draper R. C., Martin L. W., Beare P. A., and Lamont I. L. (2011) Differential proteolysis of sigma regulators controls cell-surface signalling in *Pseudomonas aeruginosa*. Mol. Microbiol. 82, 1444–1453 10.1111/j.1365-2958.2011.07901.x 22040024

[7] Enz S., Mahren S., Menzel C., and Braun V. (2003) Analysis of the ferric citrate transport gene promoter of *Escherichia coli*. J. Bacteriol. 185, 2387–2391 10.1128/JB.185.7.2387-2391.2003 12644513 PMC151517

[8] Bastiaansen K. C., Ibañez A., Ramos J. L., Bitter W., and Llamas M. A. (2014) The Prc and RseP proteases control bacterial cell-surface signalling activity. Environ. Microbiol. 16, 2433–2443 10.1111/1462-2920.12371 24373018

[9] Alba B. M., Leeds J. A., Onufryk C., Lu C. Z., and Gross C. A. (2002) DegS and YaeL participate sequentially in the cleavage of RseA to activate the sigma(E)-dependent extracytoplasmic stress response. Genes Dev. 16, 2156–2168 10.1101/gad.1008902 12183369 PMC186436

[10] Kim D. Y., Kwon E., Choi J., Hwang H. Y., and Kim K. K. (2010) Structural basis for the negative regulation of bacterial stress response by RseB. Protein Sci. 19, 1258–1263 10.1002/pro.393 20512978 PMC2895250

[11] Bishop T. F., Martin L. W., and Lamont I. L. (2017) Activation of a cell surface signaling pathway in *Pseudomonas aeruginosa* requires ClpP protease and new sigma factor synthesis. Front. Microbiol. 8, 2442 10.3389/fmicb.2017.02442 29312164 PMC5733041

[12] Bastiaansen K. C., Otero-Asman J. R., Luirink J., Bitter W., and Llamas M. A. (2015) Processing of cell-surface signalling anti-sigma factors prior to signal recognition is a conserved autoproteolytic mechanism that produces two functional domains. Environ. Microbiol. 17, 3263–3277 10.1111/1462-2920.12776 25581349

[13] Bastiaansen K. C., van Ulsen P., Wijtmans M., Bitter W., and Llamas M. A. (2015) Self-cleavage of the *Pseudomonas aeruginosa* cell-surface signaling anti-sigma factor FoxR occurs through an N-O acyl rearrangement. J. Biol. Chem. 290, 12237–12246 10.1074/jbc.M115.643098 25809487 PMC4424355

[14] Drozdetskiy A., Cole C., Procter J., and Barton G. J. (2015) JPred4: a protein secondary structure prediction server. Nucleic Acids Res. 43, W389–W394 10.1093/nar/gkv332 25883141 PMC4489285

[15] Tusnády G. E., and Simon I. (2001) The HMMTOP transmembrane topology prediction server. Bioinformatics 17, 849–850 10.1093/bioinformatics/17.9.849 11590105

[16] Schultz J., Milpetz F., Bork P., and Ponting C. P. (1998) SMART, a simple modular architecture research tool: identification of signaling domains. Proc. Natl. Acad. Sci. U.S.A. 95, 5857–5864 10.1073/pnas.95.11.5857 9600884 PMC34487

[17] Letunic I., Doerks T., and Bork P. (2015) SMART: recent updates, new developments and status in 2015. Nucleic Acids Res. 43, D257–D260 10.1093/nar/gku949 25300481 PMC4384020

[18] Jensen J. L., Wu Q., and Colbert C. L. (2018) NMR assignments of the N-terminal signaling domain of the TonB-dependent outer membrane transducer PupB. Biomol. NMR Assign. 12, 91–94 10.1007/s12104-017-9785-0 29071576 PMC5871555

[19] Holm L. (2019) Benchmarking fold detection by DaliLite version 5. Bioinformatics 35, 5326–5327 10.1093/bioinformatics/btz536 31263867

[20] Ferguson A. D., and Deisenhofer J. (2004) Metal import through microbial membranes. Cell 116, 15–24 10.1016/S0092-8674(03)01030-4 14718163

[21] Garcia-Herrero A., and Vogel H. J. (2005) Nuclear magnetic resonance solution structure of the periplasmic signalling domain of the TonB-dependent outer membrane transporter FecA from *Escherichia coli*. Mol. Microbiol. 58, 1226–1237 10.1111/j.1365-2958.2005.04889.x 16313612

[22] Cobessi D., Celia H., Folschweiller N., Schalk I. J., Abdallah M. A., and Pattus F. (2005) The crystal structure of the pyoverdine outer membrane receptor FpvA from *Pseudomonas aeruginosa* at 3.6 Å resolution. J. Mol. Biol. 347, 121–134 10.1016/j.jmb.2005.01.021 15733922

[23] Wirth C., Meyer-Klaucke W., Pattus F., and Cobessi D. (2007) From the periplasmic signaling domain to the extracellular face of an outer membrane signal transducer of *Pseudomonas aeruginosa*: crystal structure of the ferric pyoverdine outer membrane receptor. J. Mol. Biol. 368, 398–406 10.1016/j.jmb.2007.02.023 17349657

[24] Malki I., Simenel C., Wojtowicz H., de Amorim G. C., Prochnicka-Chalufour A., Hoos S., Raynal B., England P., Chaffotte A., Delepierre M., Delepelaire P., and Izadi-Pruneyre N. (2014) Interaction of a partially disordered antisigma factor with its partner, the signaling domain of the TonB-dependent transporter HasR. PloS One 9, e89502 10.1371/journal.pone.0089502 24727671 PMC3984077

[25] Härle C., Kim I., Angerer A., and Braun V. (1995) Signal transfer through three compartments: transcription initiation of the *Escherichia coli* ferric citrate transport system from the cell surface. EMBO J. 14, 1430–1438 10.1002/j.1460-2075.1995.tb07129.x 7729419 PMC398229

[26] Ferguson A. D., Amezcua C. A., Halabi N. M., Chelliah Y., Rosen M. K., Ranganathan R., and Deisenhofer J. (2007) Signal transduction pathway of TonB-dependent transporters. Proc. Natl. Acad. Sci. U.S.A. 104, 513–518 10.1073/pnas.0609887104 17197416 PMC1760641

[27] Sheffield P., Garrard S., and Derewenda Z. (1999) Overcoming expression and purification problems of RhoGDI using a family of “Parallel” expression vectors. Protein Expr. Purif. 15, 34–39 10.1006/prep.1998.1003 10024467

[28] Sasse J., and Gallagher S. R. (1991). Staining proteins in gels, p. 10.6.1–10.6.8. In Current protocols in molecular biology (Ausubel F. M., Brent R., Kingston R. E., Moore D. D., Seidman J. G., Smith J. A., and Struhl K. ed.) vol. 2, Greene Publishing and Wiley-Interscience, New York, NY

[29] Van Duyne G. D., Standaert R. F., Karplus P. A., Schreiber S. L., and Clardy J. (1993) Atomic structures of the human immunophilin FKBP-12 complexes with FK506 and rapamycin. J. Mol. Biol. 229, 105–124 10.1006/jmbi.1993.1012 7678431

[30] Doublie S. (1997) Preparation of selenomethionyl proteins for phase determination. Methods Enzymol. 276, 523–530 10.1016/S0076-6879(97)76075-0 9048379

[31] Sreerama N., Venyaminov S. Y., and Woody R. W. (2001) Analysis of protein circular dichroism spectra based on the tertiary structure classification. Anal. Biochem. 299, 271–274 10.1006/abio.2001.5420 11730356

[32] Scheuermann T. H., Stroud D., Sleet C. E., Bayeh L., Shokri C., Wang H., Caldwell C. G., Longgood J., MacMillan J. B., Bruick R. K., Gardner K. H., and Tambar U. K. (2015) Isoform-selective and stereoselective inhibition of hypoxia inducible factor-2. J. Med. Chem. 58, 5930–5941 10.1021/acs.jmedchem.5b00529 26226049

[33] Brautigam C. A. (2015) Fitting two- and three-site binding models to isothermal titration calorimetric data. Methods 76, 124–136 10.1016/j.ymeth.2014.11.018 25484338 PMC4591754

[34] Brautigam C. A., Zhao H., Vargas C., Keller S., and Schuck P. (2016) Integration and global analysis of isothermal titration calorimetry data for studying macromolecular interactions. Nat. Protoc. 11, 882–894 10.1038/nprot.2016.044 27055097 PMC7466939

[35] Zhao H., Piszczek G., and Schuck P. (2015) SEDPHAT: a platform for global ITC analysis and global multi-method analysis of molecular interactions. Methods 76, 137–148 10.1016/j.ymeth.2014.11.012 25477226 PMC4380758

[36] Zhao H., and Schuck P. (2015) Combining biophysical methods for the analysis of protein complex stoichiometry and affinity in SEDPHAT. Acta Crystallogr D Biol. Crystallogr. 71, 3–14 10.1107/S1399004714010372 25615855 PMC4304681

[37] Houtman J. C., Brown P. H., Bowden B., Yamaguchi H., Appella E., Samelson L. E., and Schuck P. (2007) Studying multisite binary and ternary protein interactions by global analysis of isothermal titration calorimetry data in SEDPHAT: application to adaptor protein complexes in cell signaling. Protein Sci. 16, 30–42 10.1110/ps.062558507 17192587 PMC1794685

[38] Vonrhein C., Flensburg C., Keller P., Sharff A., Smart O., Paciorek W., Womack T., and Bricogne G. (2011) Data processing and analysis with the autoPROC toolbox. Acta Crystallogr. D Biol. Crystallogr. 67, 293–302 10.1107/S0907444911007773 21460447 PMC3069744

[39] Evans P. (2006) Scaling and assessment of data quality. Acta Crystallogr. D Biol. Crystallogr. 62, 72–82 16369096 10.1107/S0907444905036693

[40] Leslie A. G. W. (1992) Jnt CCP4/ESF-EACMB Newslett. Protein Crystallogr. 26, 27–33

[41] Kabsch W. (2010) Xds. Acta Crystallogr. D Biol. Crystallogr. 66, 125–132 20124692 10.1107/S0907444909047337 PMC2815665

[42] Evans P. R. (1997) Jnt CCP4/ESF-EACMB Newslett. Protein Crystallogr. 33, 22–24

[43] Otwinowski Z., and Minor W. (1997) Processing of x-ray diffraction data collected in oscillation mode. Methods Enzymol. 276, 307–326 10.1016/S0076-6879(97)76066-X 27754618

[44] Terwilliger T. C., Adams P. D., Read R. J., McCoy A. J., Moriarty N. W., Grosse-Kunstleve R. W., Afonine P. V., Zwart P. H., and Hung L. W. (2009) Decision-making in structure solution using Bayesian estimates of map quality: the PHENIX AutoSol wizard. Acta Crystallogr. D Biol. Crystallogr. 65, 582–601 10.1107/S0907444909012098 19465773 PMC2685735

[45] Terwilliger T. C., Grosse-Kunstleve R. W., Afonine P. V., Moriarty N. W., Zwart P. H., Hung L. W., Read R. J., and Adams P. D. (2008) Iterative model building, structure refinement and density modification with the PHENIX AutoBuild wizard. Acta Crystallogr. D Biol. Crystallogr. 64, 61–69 10.1107/S090744490705024X 18094468 PMC2394820

[46] Adams P. D., Afonine P. V., Bunkóczi G., Chen V. B., Davis I. W., Echols N., Headd J. J., Hung L. W., Kapral G. J., Grosse-Kunstleve R. W., et al (2010) PHENIX: a comprehensive Python-based system for macromolecular structure solution. Acta Crystallogr. D Biol. Crystallogr. 66, 213–221 10.1107/S0907444909052925 20124702 PMC2815670

[47] Emsley P., and Cowtan K. (2004) Coot: model-building tools for molecular graphics. Acta Crystallogr. D Biol. Crystallogr. 60, 2126–2132 10.1107/S0907444904019158 15572765

[48] McCoy A. J., Grosse-Kunstleve R. W., Adams P. D., Winn M. D., Storoni L. C., and Read R. J. (2007) Phaser crystallographic software. J. Appl. Crystallogr. 40, 658–674 10.1107/S0021889807021206 19461840 PMC2483472

[49] Painter J., and Merritt E. A. (2006) Optimal description of a protein structure in terms of multiple groups undergoing TLS motion. Acta Crystallogr. D Biol. Crystallogr. 62, 439–450 10.1107/S0907444906005270 16552146

[50] Chen V. B., Arendall W. B. 3rd, Headd J. J., Keedy D. A., Immormino R. M., Kapral G. J., Murray L. W., Richardson J. S., and Richardson D. C. (2010) MolProbity: all-atom structure validation for macromolecular crystallography. Acta Crystallogr. D Biol. Crystallogr. 66, 12–21 10.1107/S0907444909042073 20057044 PMC2803126

[51] Krissinel E., and Henrick K. (2007) Inference of macromolecular assemblies from crystalline state. J. Mol. Biol. 372, 774–797 10.1016/j.jmb.2007.05.022 17681537

[52] Brünger A. T., Adams P. D., Clore G. M., DeLano W. L., Gros P., Grosse-Kunstleve R. W., Jiang J.-S., Kuszewski J., Nilges M., Pannu N. S., Read R. J., Rice L. M., Simonson T., and Warren G. L. (1998) Crystallography & NMR System: a new software suite for macromolecular structure determination. Acta Crystallogr. D Biol. Crystallogr. 54, 905–921 10.1107/S0907444998003254, 10.1107/S0108767398011465 9757107

[53] Franke D., Petoukhov M. V., Konarev P. V., Panjkovich A., Tuukkanen A., Mertens H. D. T., Kikhney A. G., Hajizadeh N. R., Franklin J. M., Jeffries C. M., and Svergun D. I. (2017) ATSAS 2.8: a comprehensive data analysis suite for small-angle scattering from macromolecular solutions. J. Appl. Crystallogr. 50, 1212–1225 10.1107/S1600576717007786 28808438 PMC5541357

[54] Konarev P. V., Volkov V. V., Sokolova A. V., Koch M. H. J., and Svergun D. I. (2003) PRIMUS: a Windows PC-based system for small-angle scattering data analysis. J. Appl. Crystallogr. 36, 1277–1282 10.1107/S0021889803012779

[55] Semenyuk A. V., and Svergun D. I. (1991) Gnom: a program package for small-angle scattering data-processing. J. Appl. Crystallogr. 24, 537–540 10.1107/S002188989100081X PMC4233345 25484842

[56] Piiadov V., Ares de Araújo E., Oliveira Neto M., Craievich A. F., and Polikarpov I. (2019) SAXSMoW 2.0: online calculator of the molecular weight of proteins in dilute solution from experimental SAXS data measured on a relative scale. Protein Sci. 28, 454–463 10.1002/pro.3528 30371978 PMC6319763

[57] Svergun D., Barberato C., and Koch M. H. J. (1995) CRYSOL: a program to evaluate x-ray solution scattering of biological macromolecules from atomic coordinates. J. Appl. Crystallogr. 28, 768–773 10.1107/S0021889895007047

[58] Emekli U., Schneidman-Duhovny D., Wolfson H. J., Nussinov R., and Haliloglu T. (2008) HingeProt: automated prediction of hinges in protein structures. Proteins 70, 1219–1227 17847101 10.1002/prot.21613

[59] Schneidman-Duhovny D., Hammel M., Tainer J. A., and Sali A. (2016) FoXS, FoXSDock and MultiFoXS: single-state and multi-state structural modeling of proteins and their complexes based on SAXS profiles. Nucleic Acids Res. 44, W424–W429 10.1093/nar/gkw389 27151198 PMC4987932

[60] Tria G., Mertens H. D., Kachala M., and Svergun D. I. (2015) Advanced ensemble modelling of flexible macromolecules using X-ray solution scattering. IUCrJ. 2, 207–217 10.1107/S205225251500202X 25866658 PMC4392415

[61] Valentini E., Kikhney A. G., Previtali G., Jeffries C. M., and Svergun D. I. (2015) SASBDB, a repository for biological small-angle scattering data. Nucleic Acids Res. 43, D357–D363 10.1093/nar/gku1047 25352555 PMC4383894

