# Supplementary material for: Structural basis of cell-surface signaling by a conserved sigma regulator in Gram-negative bacteria
Source: J Biol Chem. 2020 Feb 26;295(17):5795–806. doi: 10.1074/jbc.RA119.010697 (PMC7186176; doi:10.1074/jbc.RA119.010697)
Supplement: Supporting Information [file supp_295_17_5795__index.html]

Structural basis of cell surface signaling by a conserved sigma regulator in Gram-negative bacteria — Structural basis: cell surface signaling — Structural basis of cell-surface signaling by a conserved sigma regulator in Gram-negative bacteria — Structural basis of cell-surface signaling — Supporting Information 

# Structural basis of cell-surface signaling by a conserved sigma regulator in Gram-negative bacteria

## Supporting Information

- Supporting Information (to be published online) - Supporting Information Tables S1, S2, and S3 and Supporting Information Figures S1 - S6.
