# Supplementary material for: Structural basis of cell-surface signaling by a conserved sigma regulator in Gram-negative bacteria
Source: J Biol Chem. 2020 Feb 26;295(17):5795–806. doi: 10.1074/jbc.RA119.010697 (PMC7186176; doi:10.1074/jbc.RA119.010697)
Supplement: Supporting Information [file supp_RA119.010697_155285_2_supp_480275_q66nq9.pdf]

Supplemental Information

Table S1. Buried surface area per residue at the CCSSD CJM:STN interface.

CJM residue	% buried
I120	39.4
G122	30.4
Q123	19.2
N137	100.0
T138	97.0
D139	10.8
G158	23.7
E159	62.2
R192	64.1
H194	6.6
**Linker**	
A246	64.0
W247	65.9
Q249	9.4
G250	81.8
**STN residue**	
M251	57.7
L252	24.0
V253	21.7
E264	10.3
D265	42.5
R268	69.5
Y269	81.5
L291	52.1

Table S2. Summary of hydrogen bonds stabilizing the PupB NTSD:PupR CCSSD interaction.

PupB NTSD	H-bond Length (Å)	PupR CCSSD
L75-N	2.9	T288-O
L75-N	3.8	T288-OG
L75-O	2.9	T288-N
I73-O	2.9	T288-OG
S76-OG	3.5	A300-O
S76-OG	2.7	T304-OG
T77-N	3.5	S286-OG
T79-OG	2.7	S286-OG
T79-OG	3.6	S286-N

Table S3. SEC-SAXS data collection and structural parameters.

	PupR CCSSD	PupR CCSSD:PupB NTSD
**Data collection parameters**		
Instrument	BioCAT ID-18	BioCAT ID-18
Beam geometry (H μm × V μm)	5 x 3	5 x 3
Detector distance (m)	3.5	3.5
Detector	Pilatus 3 1M	Pilatus 3 1M
Wavelength (Å)	1.03	1.03
q range (Å^{-1})[a]	0.004 – 0.36	0.004 – 0.36
Exposure time (sec/frame)	1	1
Total number of frames	1100	1100
Temperature (K)	298	298
**SEC parameters**		
SEC column	Superdex 200 Increase 10/300	Superdex 200 Increase 10/300
Buffer	25 mM HEPES pH 7.5, 400 mM LiCl, 10% v/v glycerol	25 mM HEPES pH 7.5, 400 mM LiCl, 10% v/v glycerol
Sample concentration (μM)	400	800
Injection volume (μL)	500	500
Flow rate (mL/min)	0.6	0.6
**Structural parameters**		
I(0) (cm^{-1}/absorbance) (from Guinier)	17.43 ± 0.06	45.53 ± 0.24
Rg (Å) (from Guinier)	22.06 ± 0.99	26.21 ± 0.22
R^2	0.9931	0.977
I(0) (cm^{-1}/absorbance) [from *P(r)*]	17.45 ± 0.04	44.80 ± 0.18
Rg (Å) [from *P(r)*]	22.31 ± 0.06	25.81 ± 0.14
qRg range (Å^{-1})	0.58 - 1.29	0.64 – 1.29
D_{max} (Å)	75.0	87.3
χ^2 (total estimate from GNOM)	1.386 (0.835)	1.358 (0.895)
Porod volume estimate (Å^3)	48580	55800
SASBDB ID	SASDGA5	SASDGU5
**SAXS-derived molecular mass**		
From Porod volume (V_{Porod}/1.7) (kDa)	28.6	32.8
SAXSMoW2 (kDa)	23.3	40.0
Molecular mass from sequence (kDa)	24.1	32.2

**Curve fitting with atomic model**	CCSSD structure	Model 1	Model 2	Model 3	CCSSD structure	Complex structure
q range for modeling	0.026 - 0.300	0.026 - 0.300	0.026 - 0.300	0.026 - 0.300	0.024 – 0.300	0.024 – 0.300
χ^2, *P*-value	2.62, 0.00	2.16, 0.00	1.72, 0.00	2.01, 0.00	11.34, 0.00	2.45, 0.00
Predicted *Rg* (Å)	21.36	20.86	20.95	21.81	21.42	23.49
Dro (e/Å^3)[b]	0.075	0.045	0.075	0.018	0.075	0.075
Ra (Å)[c]	1.620	1.800	1.400	1.400	1.400	1.540
Vol (Å^3)[d]	31631	30013	31597	34096	31631	42276

***Ab initio* modeling**	PupR CCSSD	PupR CCSSD:PupB NTSD
q max (Å^{-1})	0.27	0.26
Number of repetitions	20	20
Normalized spatial discrepancy	0.598 ± 0.02	0.592 ± 0.02

[a] $q = 4\pi\sin(\theta)/\lambda$, where 2θ is the scattering angle.
[b] Contrast of hydration shell.
[c] Atomic group radius.
[d] Optimal excluded volume.

Supplemental Figures

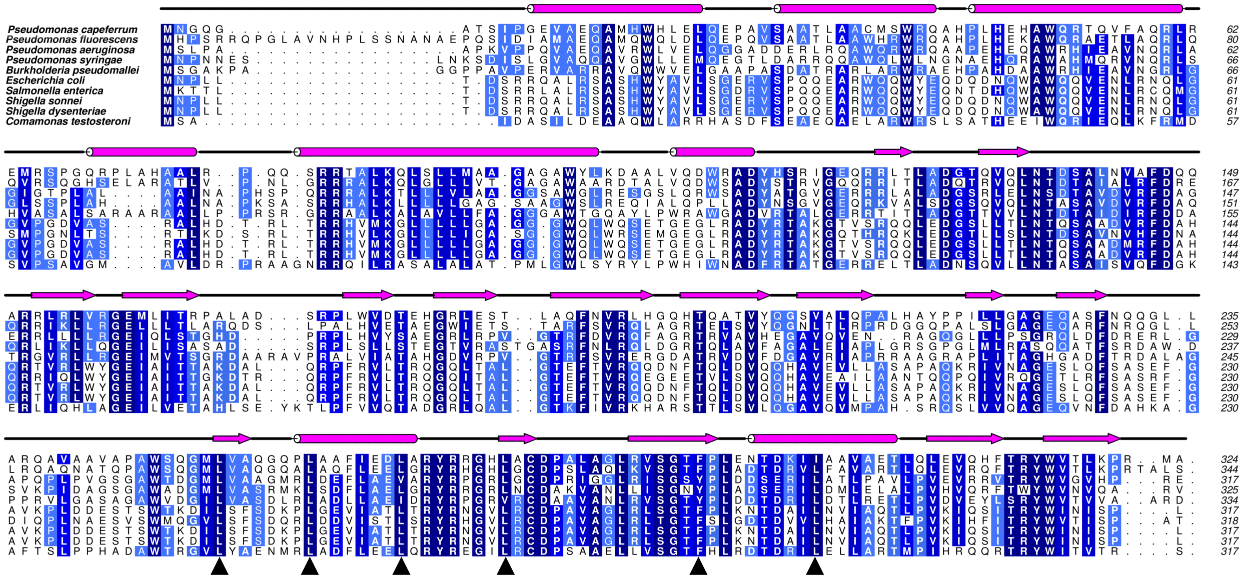

Fig S1. Sequence alignment and consensus secondary structure prediction of sigma regulator proteins from various proteobacteria with putative iron transport TBDTs. PupR (Uniprot ID Q52209, *Pseudomonas capeferrum*) was aligned with PupR (Q4KDP8*, Pseudomonas fluorescens*), FpvR (Q91192, *Pseudomonas aeruginosa*), FecR/PupR (Q48ML1, *Pseudomonas syringae*), FecR (Q63LI7, *Burkholderia pseudomallei*), FecR (P23485, *Escherichia coli*), FecR (A0A3V6D9V0, *Salmonella enterica*), FecR (Q3YU71*, Shigella sonnei*), FecR (B3WZ95, *Shigella dysenteriae*), and FecR (H1RKR8, *Comamonas testosteroni*). Invariant residues are highlighted in navy blue, and conserved residues in varying lighter shades of blue, with the lightest shade corresponding to the least conserved residues. Predicted secondary structure is displayed above the primary sequence, with cylinders, arrows and lines denoting helices, β-strands and coils, respectively. Conserved residues that stabilize the core of the PupR CCSSD STN and have previously been called the "LLLV" region are indicated (black triangles).

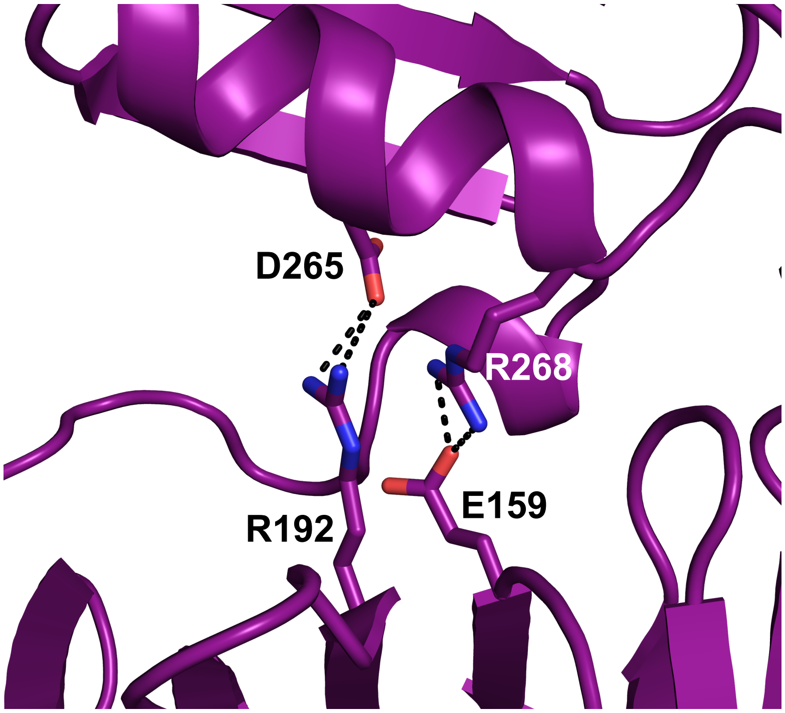

Fig S2. Salt bridges stabilizing the interface between the CJM and STN subdomains. Residues involved in salt bridges are shown in stick and colored as in Fig 2.

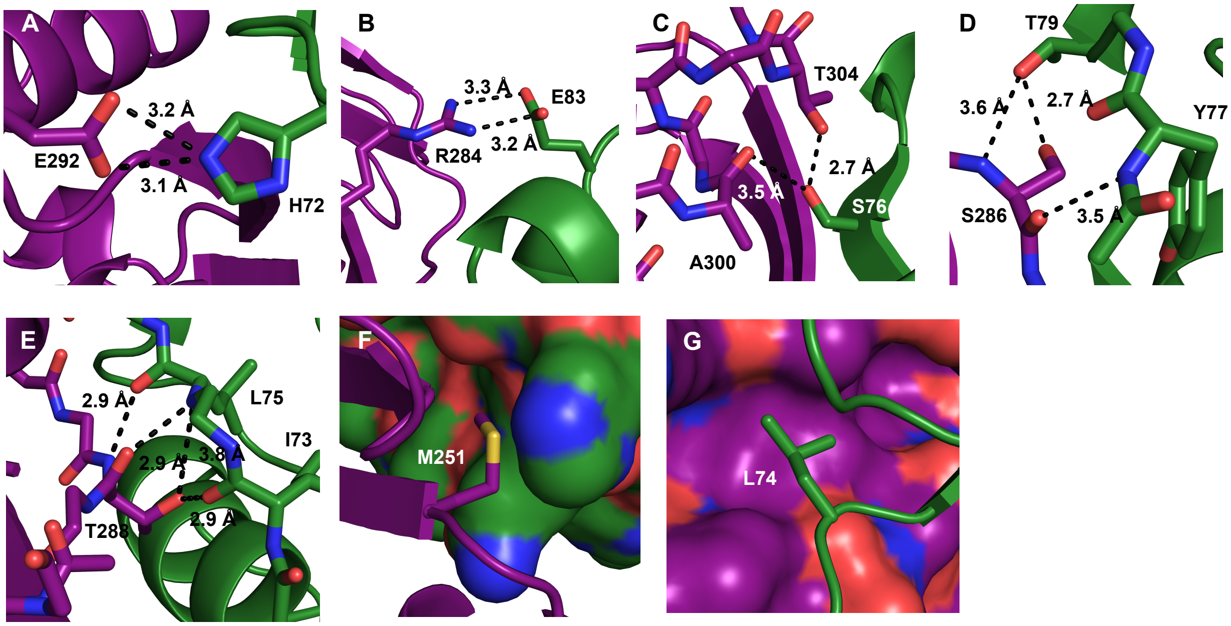

Fig S3. Interactions stabilizing the PupR CCSSD:PupB NTSD interface. Salt bridges between (A) NTSD H72 and CCSSD E292, (B) NTSD E83 and CCSSD R284; hydrogen bonding between (C) NTSD S76 and the CCSSD, (D) CCSSD S286 and the NTSD, (E) CCSSD T288 and the NTSD; and hydrophobic packing between (F) CCSSD M251 and the NTSD and (G) NTSD L74 and the CCSSD. Residues are shown in stick and colored as in Fig 2. Hydrogen bonds or salt bridges are denoted by dashes.

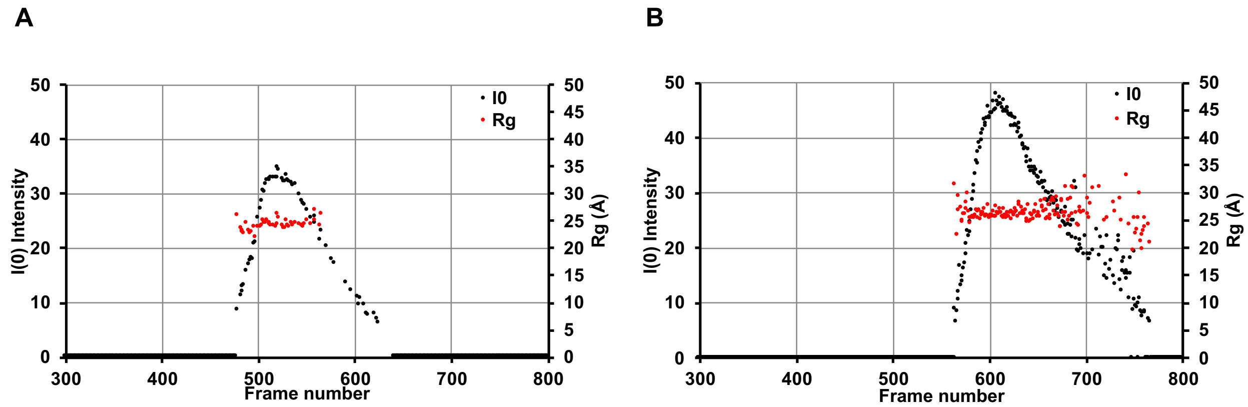

Fig S4. SEC-SAXS analysis of the concentration dependence of R_g for the (A) CCSSD and the (B) CCSSD:NTSD complex. R_g (red) and *I(0)* (black) plotted by frame number. For each, the R_g remains consistent with changing protein concentrations.

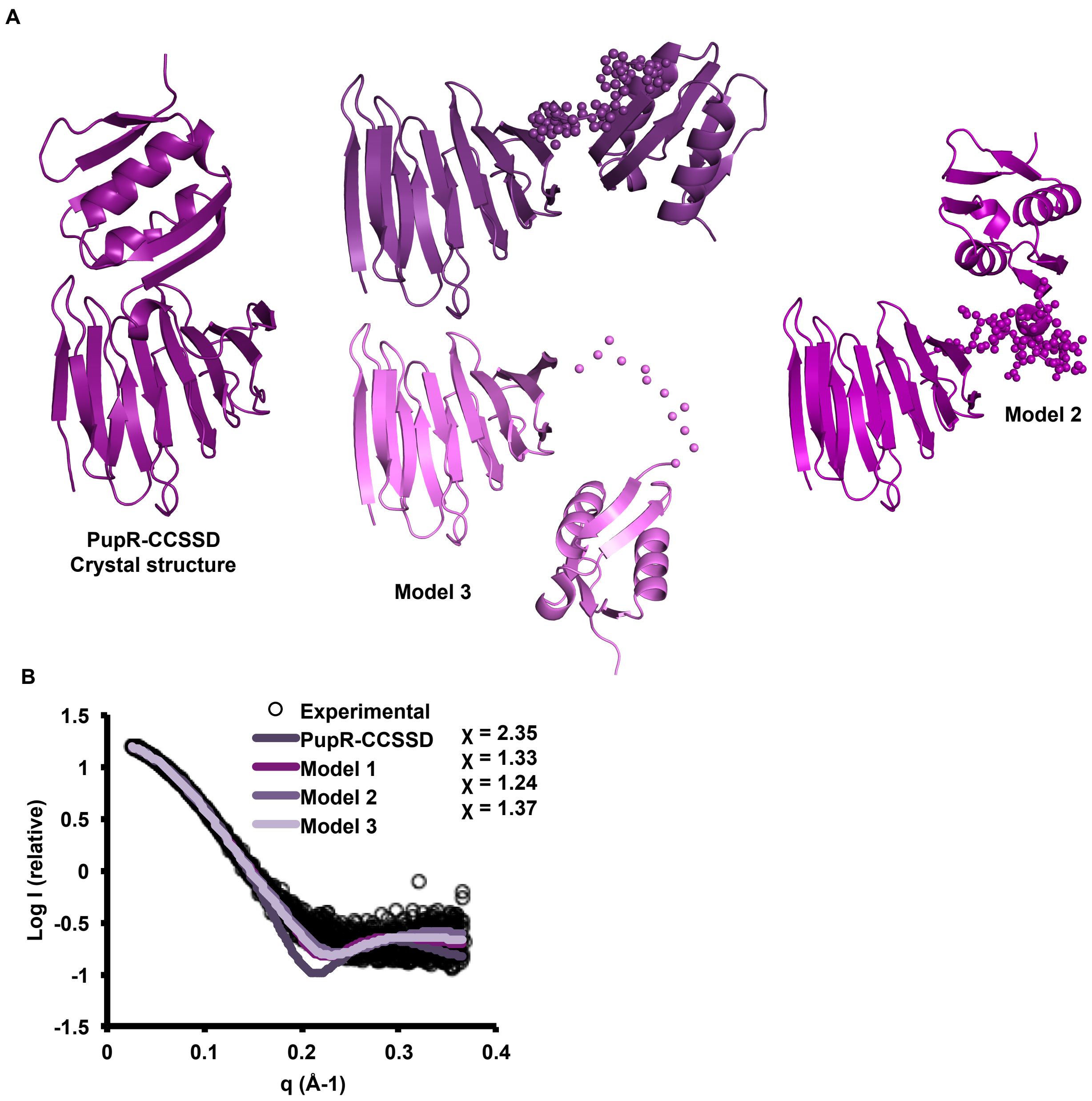

Fig S5. Conformational heterogeneity of the PupR CCSSD in solution. (A) Three PupR CCSSD models were assessed and compared with the CCSSD crystal structure: Model 1, which defined the linker region as residues 239-250; Model 2, which defined the linker region as residues 232-250; and Model 3, the EOM output. The CJM domain of each model is shown in a superimposable orientation with that of the PupR CCSSD crystal structure. The two subdomains of the CCSSD are shown in shades of purple ribbon, with linker regions of Models 1, 2, and 3 displayed as non-bonded spheres. (B) Experimental scattering profile of the CCSSD (black circles), fit with theoretical scattering profiles calculated from the CCSSD crystal structure, and Models 1-3, generated by structural conformation sampling. Chi values for each fit are indicated.

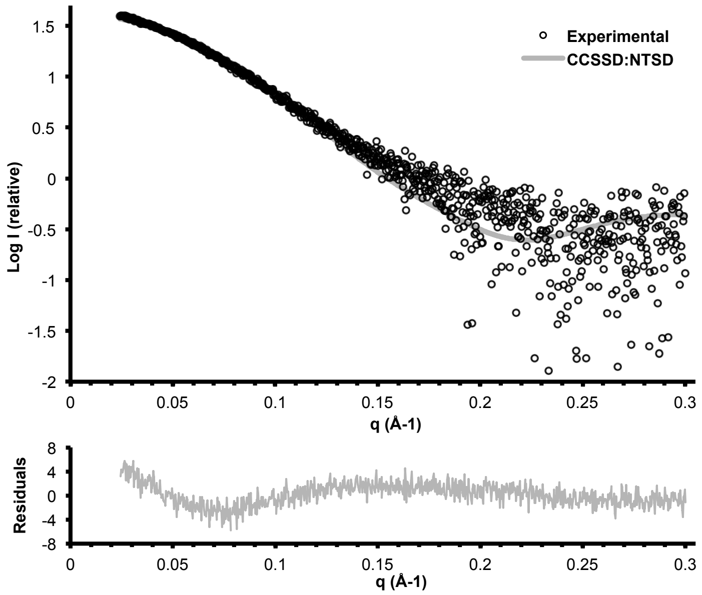

Fig S6. Experimental scattering profile of the CCSSD:NTSD complex and the fit to the theoretical scattering curve of the CCSSD:NTSD crystal structure. Top panel shows the experimental scattering profile (black circles) and the fit of the theoretical scattering profile calculated from the CCSSD:NTSD crystal structure (gray line). Bottom panel shows the plot of the residuals of the fit (gray) to the experimental scattering curve.
